# Developmental and foliation changes due to dysregulation of adenosine kinase in the cerebellum

**DOI:** 10.1038/s41598-023-47098-5

**Published:** 2023-11-14

**Authors:** Hoda M. Gebril, Tho Lai, Denise E. Fedele, Amir Wahba

**Affiliations:** 1 Departement of Biomedical Engineering, School of Engineering, Rutgers University, Piscataway, NJ 08854 USA; 2 Department of Neurosurgery, Robert Wood Johnson Medical School, Rutgers University, Piscataway, NJ 08854 USA; 3 Chemistry Department, Faculty of Science, Damietta University, New Damietta City, 34518 Egypt

**Keywords:** Biochemistry, Developmental biology, Diseases, Neurology

## Abstract

Adenosine kinase (ADK), the major adenosine-metabolizing enzyme, plays a key role in brain development and disease. In humans, mutations in the *Adk* gene have been linked to developmental delay, stunted growth, and intellectual disability. To better understand the role of ADK in brain development, it is important to dissect the specific roles of the two isoforms of the enzyme expressed in the cytoplasm (ADK-S) and cell nucleus (ADK-L). We, therefore, studied brain development in Adk-tg transgenic mice, which only express ADK-S in the absence of ADK-L throughout development. In the mutant animals, we found a reduction in the overall brain, body size, and weight during fetal and postnatal development. As a major developmental abnormality, we found a profound change in the foliation pattern of the cerebellum. Strikingly, our results indicated aberrant Purkinje cells arborization at P9 and accelerated cell death at P6 and P9. We found defects in cerebellar cell proliferation and migration using a bromodeoxyuridine (BrdU)-based cell proliferation assay at postnatal day 7. Our data demonstrate that dysregulation of ADK expression during brain development profoundly affects brain growth and differentiation.

## Introduction

The purine ribonucleoside adenosine is an ancient evolutionary regulator of energy homeostasis and controls major physiological functions ranging from biochemical pathways to brain development^1–7^. Adenosine levels in the brain are largely under the control of its main metabolic enzyme adenosine kinase (ADK), which exists in a short cytoplasmic isoform (ADK-S) and a long nuclear isoform (ADK-L)^7–9^. Whereas the cytoplasmic isoform ADK-S controls intra- and extracellular adenosine levels and hence adenosine receptor activation^5,10^, ADK-L in the cell nucleus controls the flux of methyl groups through DNA transmethylation reactions and thereby assumes a recently identified role as epigenetic regulator^11–13^. Dysregulation of adenosine metabolism is implicated in many brain pathologies, including epilepsy and congenital disorders ^6,14^.

During early developmental processes, ADK expression undergoes coordinated changes in the brain, which include a gradual transition of ADK-L dominance to ADK-S dominance and a shift from dominant expression in neural stem and precursor cells and immature neurons to a dominant expression in astrocytes^15–18^. While ADK-L expression is widespread during brain development, it is important to note that ADK-L expression in the adult brain is maintained only in a subset of neurons of the cerebellum, dentate gyrus, olfactory bulb, and in astrocytes of all brain regions^16,17^, whereas ADK-S expression in astrocytes becomes a dominant characteristic feature of the mature brain^16,17^. We recently analyzed ADK expression in the developing cerebellum and found an association of ADK-L expression with cell proliferation in the external granular layer of the cerebellar cortex^17^. The tight control of the expression of ADK isoforms during brain development suggests that disrupting those processes is detrimental and might be relevant for human developmental brain disorders.

Brain development requires a series of orchestrated events through genetic and epigenetic regulation in combination with constant dynamic changes on the cellular or molecular level^19^. Growth and foliation of the cerebellum occur in synchrony at early developmental stages. Both processes are controlled by cellular and molecular cues, including anchoring centers and canonical signaling pathways. Disruption of these cues affects both the cerebellum's size and degree of foliation^20,21^. The process of cerebellar foliation is initiated at early gestational stages in rodents through the formation of anchor points on the surface of the developing cerebellum^20^. Each of these anchor points is positioned at prospective fissures, which sub-divide the cerebellum into lobes through the anterior–posterior axes. In the mammalian cerebellum, this process results in ten cardinal lobules in the vermis (I-X) and four lobules in the cerebellar hemispheres. This foliation process is presumably under tight genetic control; however, the exact genes and molecular mechanisms are yet to be determined. Changes in the proliferation of the external granule neurons (EGNs) of the cerebellar cortex have been linked to changes in cerebellar size and degree of foliation^22^. Reduced EGN proliferation in mutant mouse models because of altered sonic hedgehog (SHH) signaling is associated with cerebellar hypoplasia characterized by a simplified foliation pattern^23^. Moreover, defective Wnt signaling has been linked to the cerebellar midline phenotype seen in Joubert syndrome, a rare brain malformation characterized by underdevelopment of the cerebellum^21^. Although regulating adenosine metabolism has been implicated in brain development^17^ and cell proliferation^17,24^, little is known about its role in cerebellar development and foliation. There is a growing body of evidence that mutations in ADK are associated with developmental and neurological retardation^25–27^, however, the role of ADK regulation for cerebellar development remains unknown. Human mutations in the *Adk* gene may provide clues for our understanding of the functional implications of global ADK deficiency for brain development. In fact, human cases with mutations in the *Adk* gene are characterized by developmental delay, stunted growth, and transmethylation defects^25–27^.

Although there is clinical evidence that the global disruption of the *Adk* gene, affecting both isoforms of ADK, causes developmental delay in conjunction with a broad spectrum of neurological phenotypes and stunted growth, the relative contribution of ADK-L and ADK-S for the expression of those phenotypes is not clear. Hence, we studied the developmental phenotype of Adk-tg mice, engineered to lack ADK-L and overexpress ADK-S in the brain.

## Results

### Effect of dysregulated ADK isoform expression on gross adult brain morphology

**Figure 1:**
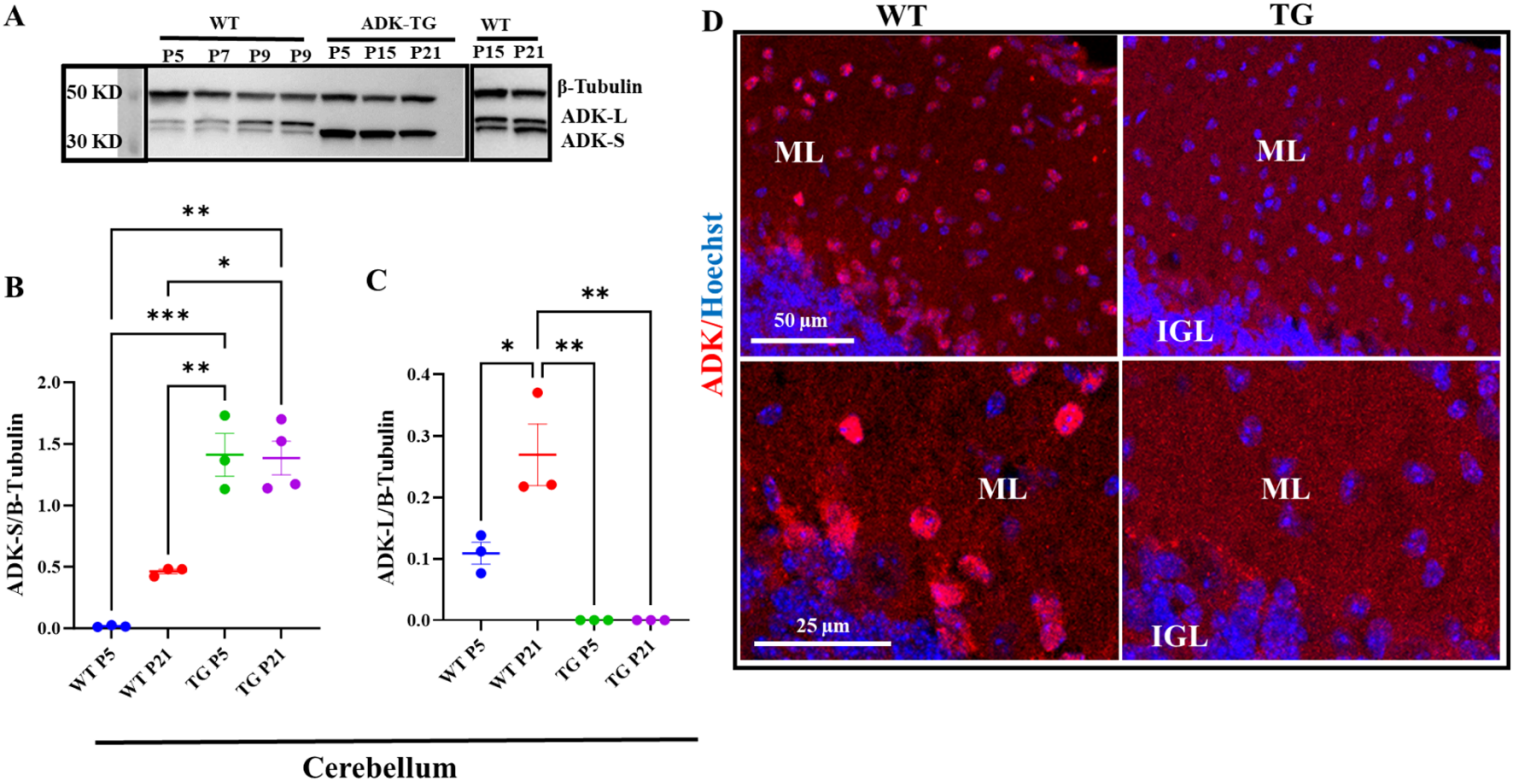
Characterization of engineered changes in the ADK expression profile during the postnatal development of Adk-tg cerebellum. (**A**) Expression profile of ADK-L and ADK-S proteins in the cerebellum from Adk-tg and WT mice at different postnatal developmental stages. Representative cropped blots show ADK (L and S) isoform expression at the cerebellar developmental stages P5, P7, P9, P15, P21, with the long ADK-L isoform appearing as an upper band and the short ADK-S isoform as the lower band. The WT cerebellum showed increased expression of both ADK isoforms (S and L) during developmental maturation. In contrast, the Adk-tg cerebellum was characterized by constant expression of the *Adk-S* transgene at all developmental stages. (**B**) Quantitative analysis of the expression profile of ADK-S in the developing cerebellum of Adk-tg and WT mice. In WT cerebellum, ADK-S expression increased progressively and significantly from P5 to P21, while constant levels (~ 1.5-fold increase of ADK-S compared to WT at P5) were found during the development of the Adk-tg cerebellum. (**C**) Quantitative analysis of the expression profile of ADK-L in the developing cerebellum of Adk-tg and WT mice. In WT cerebellum, ADK-L expression increased progressively and significantly from P5 to P21, whereas ADK-L expression was absent in the cerebellum of Adk-tg mice. Quantitative analysis was performed using Image J V. 1.52 software and expressed as the ratio of optical densities of ADK expression levels normalized to the internal standard beta-tubulin. (**D**) Immunostaining of ADK (red) and Hoechst (blue) in WT and Adk-tg mice. White arrows show colocalization of Hoechst-positive cells nuclei with Adk-L in the molecular layer (ML) and internal granular layer (IGL). Scale bar is 50 and 25 µm. All values are presented as mean ± SEM (n = 3–4). Two-way ANOVA with Tukey's multiple comparison post-hoc test (*p < 0.05, **p < 0.01, and ***p < 0.001 for significance).

**Figure 2:**
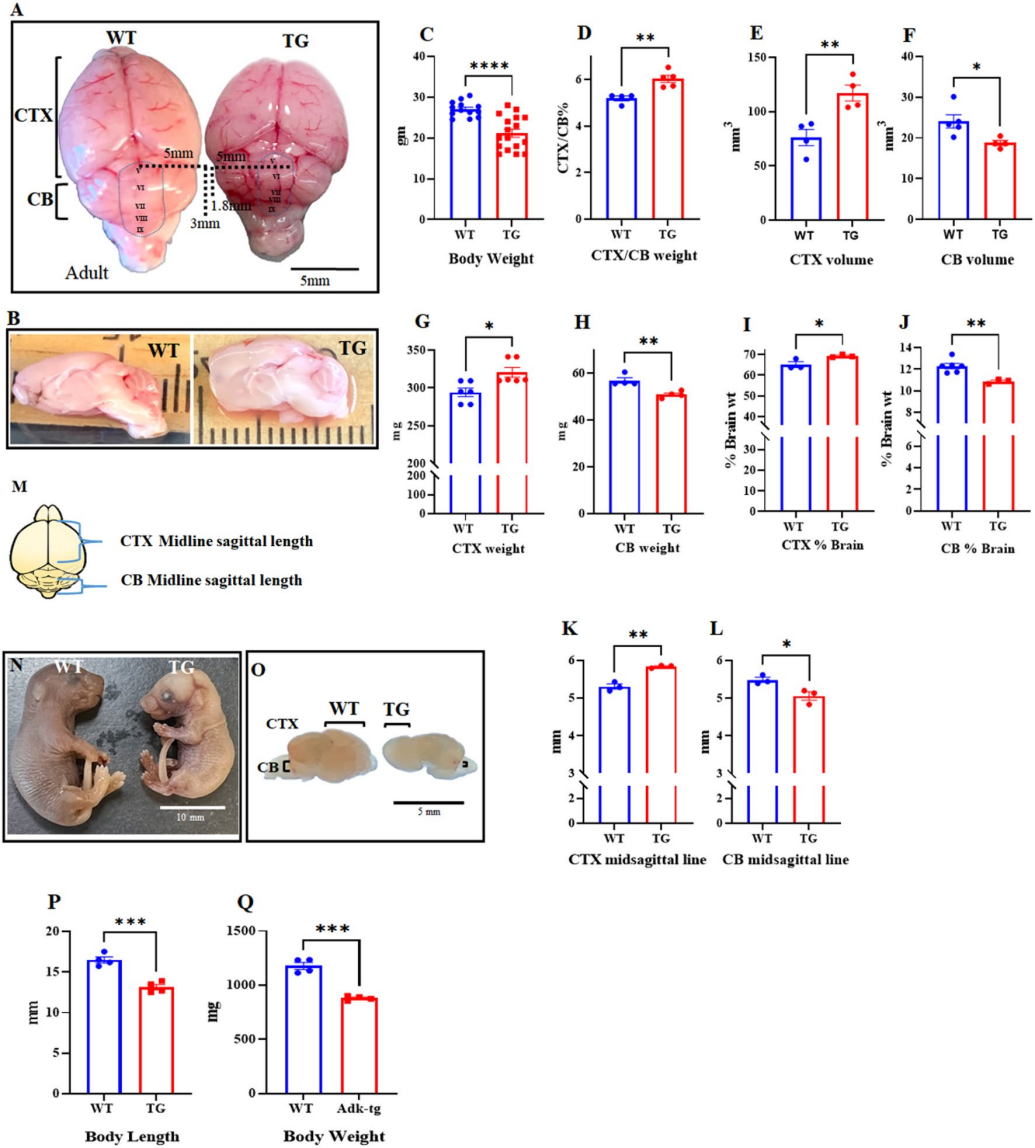
Gross morphological changes in adult and embryonic brains of Adk-tg mice. (**A**) Whole brains of an Adk-tg mouse and a WT control. (**B**) Sagittal view of Adk-tg mouse and WT control brain. (**C**) and (**D**) Statistical analysis of whole-body weight and the cortical (CTX) to cerebellar (CB) weight ratio of ADK-tg mice and WT controls. The total body weight of Adk-tg mice is significantly less than WT control. In contrast, the ratio of the cerebral cortex to cerebellum weight is significantly higher in Adk-tg compared to WT controls. (**E**) and (**F**) Statistical analysis of the volume of CTX and CB from Adk-tg mice and WT controls. In Adk-tg mice, the cerebellum volume is significantly lower, while the volume of the cerebellar cortex is significantly higher than that of WT controls. (**G**) and (**H**) Statistical analysis of CTX and CB weight in Adk-tg mice and WT controls. (**I**) and (**J**) Statistical analysis of CTX and CB weight ratio to the whole brain weight in Adk-tg mice and WT controls. In Adk-tg mice, the ratio of CTX to the total brain weight is significantly higher, while the ratio of CB weight is significantly lower than that of WT controls. (**K**) and (**L**) Statistical analysis of the length of the CTX and CB midsagittal line of Adk-tg mice and WT controls. The midsagittal line of the cerebrum in Adk-tg is longer, while it is shorter in the cerebellum than WT control. (**M**) Illustrative cartoon of the midsagittal lines of CTX and CB. (**N**) and (**O**) Whole body and whole brain images of Adk-tg and WT embryos at embryonic day E15.5. (**P**) and (**Q**) Quantitative measurements of embryonic body length and body weight. Graphs show a significant reduction in Adk-tg embryonic body length and weight. Scale bars are 10 mm and 5 mm for A, 10 mm for I, and 5 mm for J. All values are presented as mean ± SEM (n = 3–17). Unpaired 2-tailed t-test (*p < 0.05, **p < 0.01, and***p < 0.001 for significance).

ADK undergoes coordinated expression changes during brain development^16–18^ and influences cell proliferation in the brain^24^. Therefore, we predicted that dysregulation of ADK expression would lead to congenital anomalies in the brain. We used Adk-tg transgenic mice, which overexpress an Adk-S transgene within an Adk-null background and therefore lack ADK-L from birth and only express ADK-S throughout their lifespan^28^. First, using Western blot analysis, we characterized the engineered changes in the ADK expression profile during postnatal development of Adk-tg mice at day 5, 15, and 21 (P5, P15, P21) in comparison with WT mice (Fig. 1A and S1A,B). While expression of the ADK-S isoform through the transgene was stable throughout postnatal development of the Adk-tg mice (*p* = 0.008 at P5 (ADK-tg VS WT), and *p* = 0.012 at P21 (ADK-tg VS WT)), WT mice were characterized by a characteristic developmental shift of the different ADK isoforms with a dominance of ADK-L in the immature cerebellum and dominance of ADK-S in the adult cerebellum (Fig. 1B,C). Second, using immunohistochemistry, we characterized the expression of ADK in the cerebellum of Adk-tg and WT mice (Fig. 1D, S2). In WT, the expression of ADK was very intense in the nuclie of cells in the molecular layer (ML) and internal granular layer (IGL) as indicated by colocalization with the nuclear stain Hoechst. In contrast, in Adk-tg cerebellum, the expression of ADK was diffused and did not colocalize with Hoechst stain of cells nuclei. Next, we compared the gross morphology of two-month-old brains from Adk-tg mice with those from age-matched C57BL/6 wild type (WT) control mice (Fig. 2A,B). We found distinct changes in the overall morphology of the Adk-tg brain as compared to the control. The overall body weight (Fig. 2C) of ADK-tg is significantly (*p* < 0.0001) less than WT control. In particular, the cerebral cortex size was significantly enlarged (*p* = 0.0039), while the cerebellum was significantly reduced in Adk-tg mice (Fig. 2A–M). The cerebral volume, weight, the ratio of weight to the whole brain, and midsagittal length were significantly increased (*p* = 0.007, *p* = 0.012, *p* = 0.04, and *p* = 0.001, respectively) in Adk-tg mice compared to control mice (Fig. 2D,E,G,I,K). In contrast, the cerebellum volume, weight, the cerebellar midsagittal length, and the ratio of weight to the whole brain were significantly reduced (*p* = 0.02, *p* = 0.005, *p* = 0.007, and *p* = 0.007, respectively) in Adk-tg mice compared to the control (Fig. 2D,F,H,J,L). Because Adk-tg mice express a constitutively expressed transgene on top of a constitutive knock-out background, we also predicted morphological alterations during fetal development. We, therefore, examined Adk-tg mice at embryonic day 15.5 (E15.5) (Fig. 2N–Q). The body size of Adk-tg mice at E15.5 was remarkably smaller (*p* = 0.0006 and *p* = 0.0001) compared to WT (Fig. 2P& Q).

### Dysregulation of ADK expression affects the foliation pattern of the cerebellum

**Figure 3:**
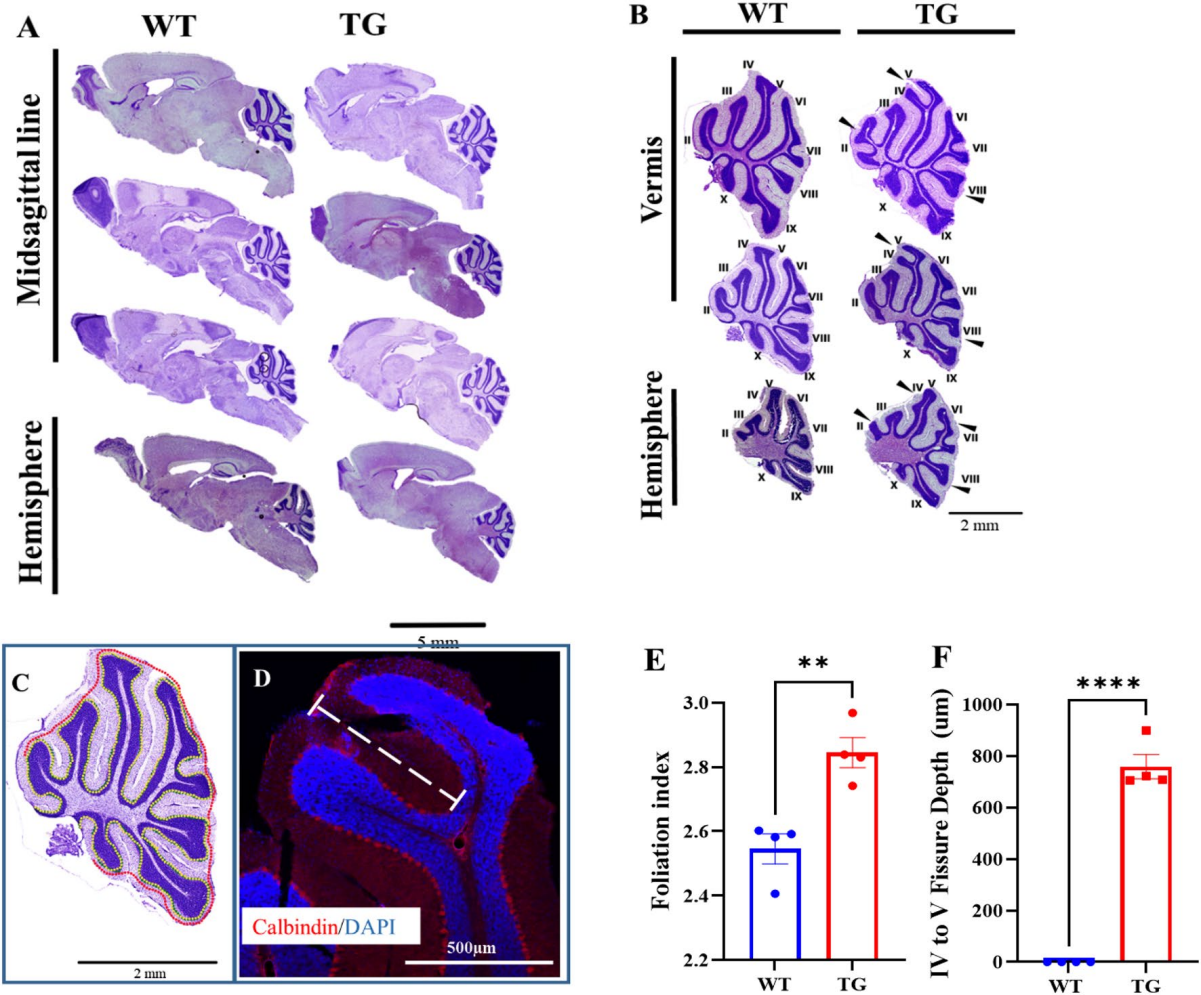
Disrupted foliation pattern in the adult cerebellum of Adk-tg mice. (**A**) Nissl-stained sagittal brain sections from the midsagittal line and the hemispheres of adult Adk-tg and WT mice. (**B**) Nissl-stained adult cerebellum from the vermis and the hemispheres of Adk-tg and WT cerebellum showing detailed differences in the CB lobules II to X. Black arrows pointed toward the extra lobules and bifurcation points in the Adk-tg cerebellum. (**C**) Adult vermis from WT CB illustrating the method used to measure the foliation index. The red dotted line represents the outline of the outermost aspects of the cerebellar folia around the Purkinje cell layer (PL). The yellow dotted line represents the tracing outline of the PL. The ratio between the length of these lines provides the foliation index. (**D**) Adult vermis from Adk-tg brain magnifying the split end at lobule IV and V. Dashed line in D illustrates the depth between lobule IV and V. (**E**) Graph representing the foliation index of Adk-tg and WT CB. (**F**) Graph representing the quantitative measurements of the depth between lobule IV and V in Adk-tg and WT CB. All values are presented as mean ± SEM (n = 4). Unpaired 2-tailed t-test (**p < 0.01 and ****p < 0.0001 for significance). Scale bar is 5 mm in A, 2 mm in B and C, and 500 µm in D.

Because the gross morphology of the Adk-tg mouse brains shows striking differences compared to WT, we investigated the cerebellum of the Adk-tg brain in more detail. First, we compared the foliation pattern in Nissl-stained adult brain sections from the midsagittal line (vermis of the cerebellum) and hemispheres and discovered striking changes in the foliation pattern of the cerebellum (Fig. 3A). In the cerebellum of all Adk-tg mice (n = 4), an additional bifurcated lobule was found after lobule II in the vermis (Fig. 3B). In addition, lobules V, VI, and VIII were characterized by split ends in the areas of the vermis and lateral cerebellum (Fig. 3B,F). We next quantified the observed changes in the foliation pattern by tracing the outline of the Purkinje cell layer of the midsagittal sections as well as the outline of the outermost aspects of the cerebellar folia around the Purkinje cell layer^29,30^ (Fig. 3C,E). The foliation index of the cerebellum of Adk-tg was significantly higher (~ 1.2 fold) (*p* = 0.0039) compared to WT (Fig. 3C,E). Moreover, using calbindin-stained brain sections, the split end between lobule IV and V was significantly observed in Adk-tg compared to WT control (*p* < 0.0001) (Fig. 3D,F)^31^.

**Figure 4:**
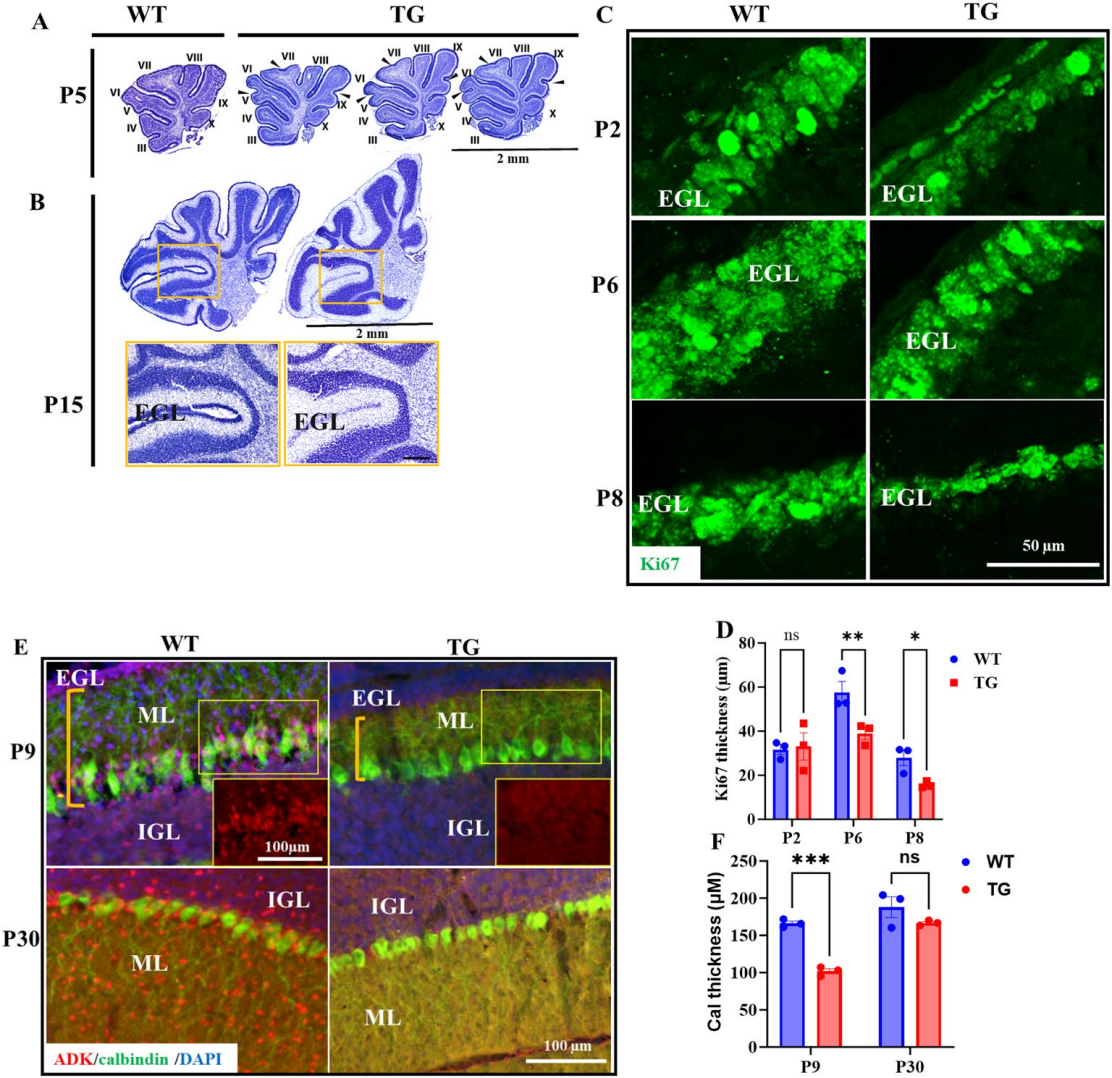
Disrupted foliation pattern in the developing cerebellum of Adk-tg mice. (**A**) Nissl-stained cerebellum of Adk-tg and WT mice at postnatal day 5 (P5). Black arrows pointed toward the bifurcation points in the vermis of the Adk-tg cerebellum. (**B**) Nissl-stained sections from Adk-tg and WT cerebellum at P15. High-magnification images of the Nissl-stained hemisphere sections from Adk-tg and WT control mice at P15 illustrate the difference in the thickness of the external granule layer (EGL) between both lines. (**C**) Immunofluorescence reactivity of Ki67 (green) in Adk-tg and WT control mice in cerebellar sections at P2, P6, and P8 illustrating changes in the thickness of EGL. (**D**) Quantitative analysis of Ki67 layer thickness in P2, P6, and P8 Adk-tg and WT mice cerebellum. (**E**) Immunofluorescence reactivity of calbindin (green) and ADK (red) in P9 and adult Adk-tg and WT control mice illustrating changes in Purkinje layer at P9 in ADK-tg. (**F**) Quantitative analysis of Purkinje layer thickness in Adk-tg and WT mice cerebellum at P9. Scale bar is 2 mm in A and B, 200 µm (yellow box) and100 µm in B, and 50 µm in C, and 100 µm in E. All values are presented as mean ± SEM (n = 3). In D, two-way ANOVA with Tukey's multiple comparison post-hoc test (*p = 0.02 and **p = 0.0013 for significance). In F, Unpaired 2-tailed t-test ****p = 0.0002 for significance).

Changes in the cerebellar foliation pattern in adult Adk-tg mice suggest a developmental defect arising from dysregulated ADK expression during brain development. We, therefore, investigated the cerebellar development at P5 and P15 in more detail. Sagittal brain sections from Adk-tg mice at postnatal day 5 and 15 (P5 and P15) were Nissl stained and compared with WT control (Fig. 4A,C). Furthermore, we found additional bifurcation at the end of lobules IV, VII, and IX in the vermis of all Adk-tg mice (n = 3) as compared to aged-matched WT mice, similar to our findings in the adult Adk-tg mice (Fig. 4A). Additional lobule was visible in whole frozen cerebellum of Adk-TG mice (Suplementary Fig. S3). In addition, the lateral sides of the cerebellum of Adk-tg mice at P15 were composed of irregular folds compared to WT. Surprisingly, the EGL in Adk-tg mice at P15, as revealed by Nissl staining, was barely detectable at this age compared to WT at P15 (Fig. 4B). Similarly, the thickness of the layer of Ki67-positive cells was significantly thinner at P6 and P8 in Adk-tg mice than WT (* = *p* = 0.02 and ** = *p* = 0.0013) (Fig. 4C,D). It is well-established that the cerebellar foliation is associated with Purkinje cells (PC) develeopment^32^. Further, ADK has been shown to play a role in Purkinje cells development^17^. Therefore, PC were stained with calbinding in cerebellum sections of ADK-tg and WT mice at P9, key developmental stage of PC development, and P30. As expected, stunted PC dendrites were observed at P9 (Fig. 4E,F) but not at P30.

### Dysregulated ADK expression is associated with deficits in neuronal cell proliferation and migration

**Figure 5:**
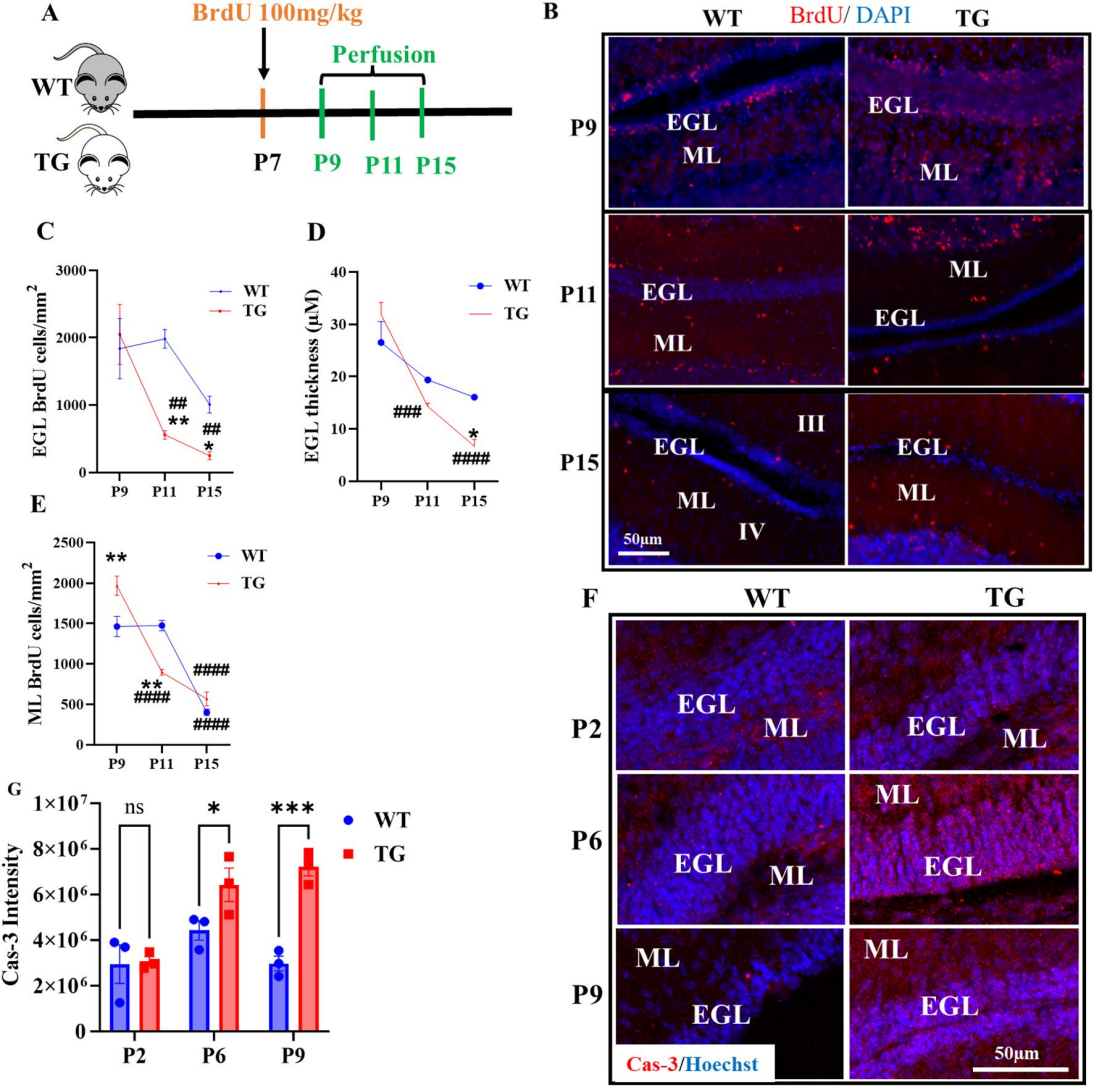
Disrupted cell expansion during the cerebellum development in Adk-tg mice. (**A**) Flowchart illustrating the BrdU injection paradigm and endpoints for sample collection. Adk-tg and WT mice at P7 were injected with BrdU 100 mg/ml, i.p. Subgroups from both genotypes were perfused at different developmental stages (P9, P11, and P15). (**B**) Immunofluorescence of BrdU (red) in the cerebellum (lobe III and IV) counter stained with DAPI (blue) at different developmental stages (P9, P11, P15). BrdU-positive cells are shown in the EGL and the molecular layer (ML). (**C**) Graph illustrating BrdU-positive cell counts per mm^2^ in the EGL at P9, P11, and P15 in Adk-tg and WT mice. (**D**) Graph illustrating the thickness of the EGL at P9, P11, and P15 in Adk-tg and WT mice. (**E**) Graph illustrating BrdU-positive cell counts per mm^2^ in the ML at P9, P11, and P15 in Adk-tg and WT mice. (**F**) Immunofluorescence reactivity of Cas-3 (red) counter stained with Hoechst in Adk-tg and WT mice at different developmental stages (P2, P6, and P8). F. Quantitative analysis of Cas-3 intensity in Adk-tg and WT mice at different developmental stages (P2, P6, and P8) illustrating changes in cell death in ADK-tg at P6 and P9. All values are presented as mean ± SEM (n = 4) in C, D, and E. Two-way ANOVA with Sidak's multiple comparison post-hoc test (*p < 0.05, and **p < 0.01 for significance). All values are presented as mean ± SEM (n = 3) in G. Two-way ANOVA with Tukey’s multiple comparison post-hoc test (*p = 0.02, and ***p = 0.0001 for significance). Scale bar is 100 µm in B. Scale bar is 100 µm in B and 50 µm in F.

The cerebellum's size and foliation depend on cell expansion in the cerebellar cortex, which requires coordination in cell proliferation, survival, migration, and differentiation^20,33,34^. To evaluate the effects of dysregulated ADK on cerebellar cell expansion, we injected Adk-tg and WT pups at P7 with BrdU (100 mg/kg), then analyzed BrdU immunohistochemistry (of mitotic and postmitotic cells) at different developmental stages (P9, P11, and P15) (Fig. 5A,B). In the EGL at P9, a similar pool of BrdU- positive cells was found in both lines (Fig. 5C). However, at P11, the number of BrdU-positive cells in the EGL was significantly reduced (*p* = 0.009) in the Adk-tg mice, representing only about 20% of the number of BrdU-positive cells in WT mice (Fig. 5C). The continued drop in the number of BrdU-positive cells in the EGL of Adk-tg mice at P11 and P15 was also associated with a reduction in the thickness of the EGL (Fig. 5D). Indeed, the differences in EGL thickness and EGL BrdU cell count were strikingly pronounced in Adk-tg mice between the developmental stages P9 and P11 as well as P9 and P15 which is not the case in WT control mice at the same developmental stages (Fig. 5C). These findings as well as the low expression of the proliferation marker, Ki67 in the EGL at P15 of Adk-tg (Fig. 4C) suggest a disruption in the cell proliferation during the cerebellum development in Adk-tg mice due to dysregulated ADK. The drop in BrdU-positive cells in the EGL of Adk-tg mice may result from accelerated migration or apoptosis of postmitotic cells^35^, which might be investigated by studying BrdU-positive cells in ML at different developmental stages^35^. At P9 in Adk-tg mice, we found a significant increase in BrdU-positive cells compared to WT mice (*p* = 0.004) suggesting an accelerated migration or reduced apoptosis throughout ML (Fig. 5E) compared to WT control. In contrast, at P11, there was a sharp reduction in the number of BrdU-positive cells in the ML of Adk-tg mice compared to WT mice (*p* = 0.001) (Fig. 5E). Eight days after the BrdU injection (P15), the number of BrdU-positive cells in the ML was reduced in both genotypes compared to P9 (Fig. 5E). These data suggest that the dysregulation in ADK expression is associated with changes in cell expansion including Ki67-positive proliferative cell layer and migration during the cerebellum development which may facilitate changes in the foliation index and cerebellar size. To further investigate whether the observed changes in EGL and ML in ADK-tg mice, cerebellar sections from ADK-tg and WT at P2, P6, and P9 were stained with the apoptotic marker Caspase-3 (Fig. 5F). As expected, accelerated cell death was significant at P6 and almost twofold at P9 in ADK-tg when compared to WT (Fig. 5F,G).

## Discussion

In this study, we tested the hypothesis that dysregulation of ADK expression affects brain growth and differentiation. Using Adk-tg transgenic mice, which overexpress ADK-S in the absence of ADK-L throughout development, we show a reduction in overall brain and body size, as well as reduced weight, during fetal and postnatal development. Importantly, we discovered changes in the foliation pattern of the developing and adult cerebellum in Adk-tg mice. Finally, we found a disruption of the cerebellar cell proliferation and migration patterns during the postnatal days 9, 11, and 15 during the development of Adk-tg mice.

### Dysregulated expression of ADK isoforms affects brain morphology

In this study, we found that dysregulated expression of ADK causes congenital changes in the brain, which is in line with prior findings showing that brain development is associated with coordinated expression changes of the ADK isoforms^16,17^. In agreement with our findings, human mutations in the *Adk* gene lead to developmental delay, stunted growth, and transmethylation defects^25–27,36^. In Fig. 2, we show that in Adk-tg mice, with constitutively expressed transgene on top of a constitutive knock-out background, the cerebellar and the cerebral size changes are differential. This may be explained by the reported differential levels of ADK isoforms during development and adulthood in the cerebellum and cerebrum^17^. Indeed, the lack of the *Adk* gene at early embryonic stages leads to postnatal death; therefore, it was not feasible to study its role in cerebellar development^37^.

In the developing WT cerebellum, nuclear ADK-L expression dominates over cytoplasmic ADK-S, while ADK-S increases at later developmental stages (Fig. 1). Here, we show that in Adk-tg cerebellum, ADK-S is consistently overexpressed in the absence of ADK-L at all developmental stages. Given the fact that ADK-L is abundant in most progenitor cells in the developing cerebellum, including granule neurons, Purkinje, and Bergman glial cells^17^, we expect that the absence of ADK-L in those cells would impact the dynamic events that control cell expansion and the size of cerebellum. Although changes in Adk-tg cerebellum can be associated with dysregulated metabolism of the extracellular adenosine due to consistent expression of ADK-S during cerebellum development, the level of release of cellular adenosine to the extracellular space at this developmental stage is undetectable^38^.

Further analysis of the cerebellum in Adk-tg mice revealed changes in the foliation pattern. The initial formation of cerebellar fissures, including the appearance of anchoring centers, was reported to be driven by the coordinated action of granule neurons and Bergmann glial cells^20^. This coordination at early developmental stages of the cerebellum was found to dictate the shape of the folia^20^. Therefore, we expect that the absence of ADK-L in the progenitors of granular neurons and Bergmann glial cells influence the foliation pattern in Adk-tg cerebellum. Indeed, given the reported role of PC development on the cerebellum foliation^39^, the observed stunted PC dendrites at the critical developmental stage (P9) may explain the aberrant cerebellar foliation in ADK-tg mice. Also, ADK was suggested to play a key role during PC development^17^. In humans, cerebellar malformations are associated with neurodevelopmental deficits^40^. In line with this notion, Adk-tg mice are characterized by altered sleep physiology^41^, severe learning deficits^42^, and develop spontaneous seizures^43^.

### Dysregulated ADK expression leads to deficits in neuronal cell proliferation and migration in the cerebellum

In this study, we demonstrated the chronical and spatial distribution of the cerebellar proliferative cells in Adk-tg cerebellum. Using a paradigm of BrdU i.p injection at P7, we found significant changes in the pattern of cell proliferation and migration in Adk-tg cerebellum. These changes include a significant reduction in the thickness of the EGL and the number of BrdU-positive cells in this layer at P11 and P15 in Adk-tg mice. At the same developmental stages, changes in EGL are associated with changes in BrdU cells in ML, which suggest either dysregulation in cell migration or apoptosis of postmitotic cells. In line with these findings, accelerated apptosis was evident in Adk-tg mice at P6 and P9.Previous studies and case reports have linked cerebellar malformation to genetic risk factors such as FOXC1^44^, CCDC22^45^. Also, mutations in genes regulate cell migration, proliferation, and microtubule formation are constitute a significant of a broad range of congenital cerebellum anomalies^46–48^. On the other hand, non-genetic risk factors have been linked to cerebellar malformation^49^. Interestingly, hemimegalencephalyde in humans has been associated with de novo mutations in genes linked to the mammalian target of rapamycin (mTOR) signaling^50^. Of note, a possible molecular link between ADK and mTOR signaling pathway has only been reported in a study on primary β-cell replication in three species (mouse, rat, and pig)^51^. Although human cases with mutations in Adk-gene are linked to developmental and neurological retardation, none has investigated the cerebellar structure in those cases^25–27^. Our findings report a link between ADK and cerebellar malformation in mice.

## Limitations

In our study, we demonstrate that dysregulation of ADK during brain development is associated with changes in cerebellar development and foliation changes. Because our mouse model overexpresses ADK-S within an Adk-null background^28^, it is not possible to determine whether the observed developmental changes are due to the overexpression of ADK-S or the lack of ADK-L.

## Conclusions

This study provides evidence that dysregulated ADK is associated with cerebellar malformation. Here, we identified a new role of ADK as a risk factor associated with developmental anomalies. In transgenic animals engineered to overexpress ADK-S over a knock-out background, we identified congenital changes in the cerebellum size and the foliation pattern. Also, we elucidated that dysregulation in ADK leads to deficits in the cerebellum's spatiotemporal pattern of proliferative cells at different developmental stages.

## Methods and materials

### Animals

All animal procedures were approved by an AAALAC-accredited facility following approved Rutgers University Institutional Animal Care and Use Committee (IACUC) protocols, in adherence to the ARRIVE guidelines, and carried out in accordance with the principles outlined in the NIH Guide for the Care and Use of Laboratory Animals. All mice were socially housed under standardized conditions of light, temperature and humidity, environmental enrichment, and had access to food and water ad libitum. The sex of prenatal, embryonic, and postnatal mice was not specified, while all adult mice used for this study were males. Adk-tg mutant mice were originally generated by transgenic overexpression of ADK-S in an Adk-null background (B6,129P3) as described^28^, then backcrossed with an Emx1Cre line in a B6 background^52^ to generate Adk-tg mice and EmxCre+/–Adk-tg mice as littermates. These backcrossed Adk-tg mice were maintained by inbreeding as a homozygous line. As a result, Adk-tg mice express only ADK-S in the absence of ADK-L. Control animals were commercial C57BL/6 mice (Jackson Lab).

### Immunohistochemistry

Mice at embryonic day 16.5 (E16.5) were collected from anesthetized dams. Mice at postnatal days 5, 15, 30, (P5–P30) as well as adult mice, were anesthetized and sacrificed by transcardial perfusion with ice-cold 4% paraformaldehyde. Brains were post-fixed for 24 h in 4%PFA. Also, following post-fixation, brains were transferred to 30%sucrose with 0.1%sodium azide in 1 X PBS for 2 days at 4 °C and stored at – 80 °C. Brains were cut sagittally into 30 µm sections on a freezing, sliding stage cryostat (Leica CM3050S). Until further processing, all adult brain sections were stored in cryoprotectants. Immunohistochemistry of the mouse brain was performed on free-floating sections. For postnatal tissues P5–P15, sagittal 30 µm sections were directly mounted on slides and then frozen until further processing. For immunofluorescence staining, antigen retrieval was performed using sodium citrate buffer pH 6 at 90 °C for 3 min, then sections were washed in 1 X TBS (Tris-Buffered Saline) and 0.05% Triton X-100 (TBS-T) three times. Then, sections were incubated at 4 °C overnight in Donkey blocking buffer containing corresponding primary antibodies. The primary antibodies used were, polyclonal goat anti- calbindin (Cal) (Abcam ab156812, 1:250), polyclonal rabbit anti-ADK (Bethyl Labs, A304-280A, 1:1000), monoclonal rat anti-Ki67 (Thermo Fisher, 14-5698-82, 1:200), monoclonal Caspase-3 Antibody (Thermo Fisher, 43-780, 1:50), and BrdU (abcam, ab6326, 1:100). Sections were washed in 1X TBS-T, then incubated for one hour at room temperature in a solution containing the corresponding secondary antibodies. Secondary antibodies included Donkey Alexa Fluor 488 (Invitrogen, A21208, 1:2000), Donkey Alexa 555 (Thermo Fisher, A-21432, 1:1000), and Donkey Alexa Fluor 633 (Invitrogen A21082, 1:250). Sections were washed three times for 5 min in 1XPBS, then mounted on slides and allowed to dry in the dark. Once dried, sections were cover-slipped with DAPI mounting medium and stored in the dark at 4 °C.

For Nissl staining, sections were mounted on slides and allowed to dry overnight. Sections were rinsed in xylene then hydrated in serial dilutions of alcohol. After incubation in 0.1%cresyl violet solution for 7 min, sections were differentiated in alcohol, cleared in xylene, and mounted with mounting medium (Fisher Scientific, SP15-100 UN1294).

### Image acquisition and cell counting

Nissl and fluorescence images were acquired using a Zeiss LSM 780 confocal microscope and a Leica microscope fitted with a StereoInvestigator system (Microbrightfield) comprising color and monochrome digital cameras. For image analysis, we selected four 30µm sections for each stain from each mouse, spaced every 150µm, and spanning the same mid-lateral location between animals (n = 3–4 animals per group). An unbiased exclusion of poor-quality images was made prior to analysis. Double-labeled cells in the EGL (BrdU/DAPI) were counted at 20 × at different focal planes in Z stacked images (n = 3/group). Cell counts were quantified in at least two to three sections per animal and presented as cell number per mm^2^. Binarization of BrdU and DAPI stained images was performed using the ImageJ software “auto threshold” command. The optimal threshold values for each stain were achieved by manually adjusting the pixel intensity range on a set of images^53^. To maintain consistentcy throughout all sections, the determined range of pixel intensities was then applied for the rest of images. Using the “particle analysis” command, the total number of cells in the region of intererest was quantified automatically.

### Volume estimation

Fresh brains were extracted directly from anesthetized decapitated animals. Brains were transsagittaly sliced into 500µm thick slices using brain matrix (Kent- RBMS). Brain sections were mounted on slides and imaged using Leica microscope. The volumes of the cerebellum and cerebrum were estimated using unbiased stereology using stereoinvistigator software (version 11.07, MBF Bioscience, Williston, VT USA). All meausrements were done using a calvarlie volume estimator function (gride size 200 µm × 200 µm, CE < 0.01). The cerebellar and cerebral volumes were sampled through the medio-lateral extent of the sagittal axis of the brain.

### Cerebellar foliation

The degree of foliation was measured as described before^29,30^. Briefly, we traced the outline of the Purkinje cell layer of the midsagittal sections as well as the outline of the outermost aspects of the cerebellar folia around the Purkinje cell layer (Fig. 4). The ratio between these two tracing lines is the degree of folding termed as cerebellar foliation index (CFI). To increase the rigor of our analysis, we analyzed the cerebellum of animals from different breeding pairs.

### Statistical analysis

The data were analyzed using Graphpad Prism software, version 8.4.3. All data, unless specified, were presented as mean ± SEM using appropriate statistical analysis (either t-test or two-way ANOVA followed by multiple comparison post-hoc test, (* = *p* ≤ 0.05 ,** = *p* ≤ 0.01, *** = *p* ≤ 0.001, **** = *p* ≤ 0.0001 for significance)).

### Supplementary Information

Supplementary Figures.

## Data Availability

All data analyzed during the current study are available from the corresponding authors on reasonable request. Also, research materials will also be made available when it is required.
