# Supplementary material for: Developmental and foliation changes due to dysregulation of adenosine kinase in the cerebellum
Source: Sci Rep. 2023 Nov 14;13:19831. doi: 10.1038/s41598-023-47098-5 (PMC10645999; doi:10.1038/s41598-023-47098-5)
Supplement: Supplementary file 1 — Supplementary Figures. [file 41598_2023_47098_MOESM1_ESM.docx]

**Supplementary materials to Research Article for Developmental Biology**

**Developmental and foliation changes due to dysregulation of adenosine kinase in the cerebellum**

Hoda M. Gebril^1*^, Tho Lai^2^, Denise E. Fedele^2^, and Amir Wahba^2,3^

*^1^Departement of Biomedical Engineering, School of Engineering, Rutgers University, Piscataway, NJ 08854, USA*

*^2^Department of Neurosurgery, Robert Wood Johnson Medical School, Rutgers University,*

*Piscataway, NJ 08854, USA*

*^3^Chemistry Department, Faculty of Science, Damietta University, New Damietta City, 34518,*

*Egypt*

**Running title: Adenosine kinase and cerebellum foliation**

*Address of correspondence: Hoda Gebril, PhD, Department of Biomedical Engineering, School of Engineering, Rutgers University, Piscataway, NJ08854, USA

Tel.: +1 (848) 445-6593; email: Hoda.Gebril@rutgers.edu


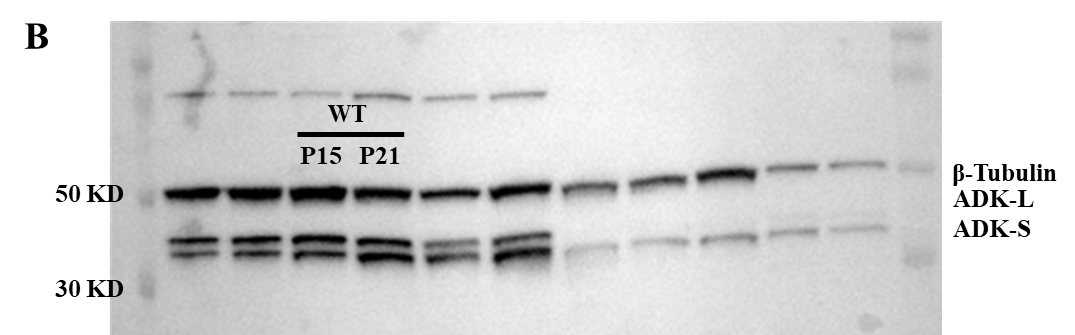

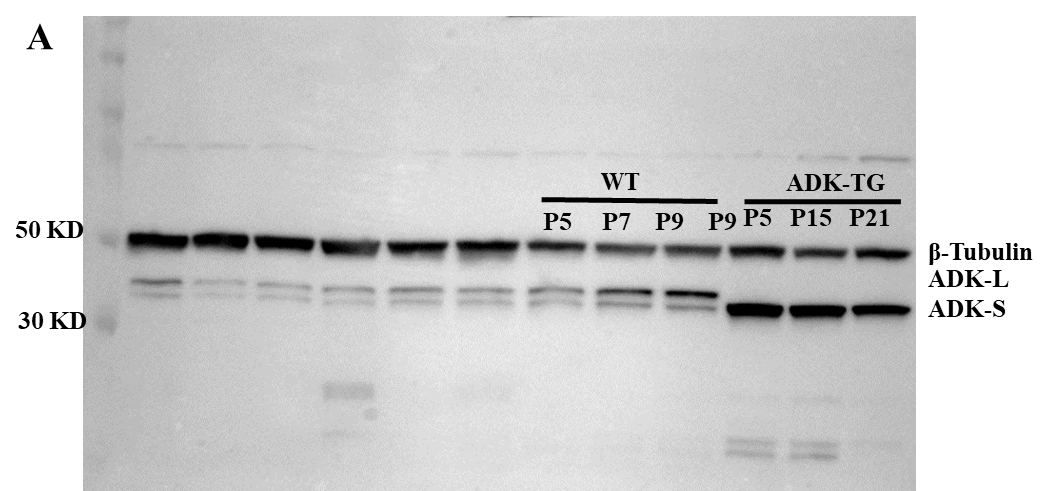


**Supplementary Figure** **1 (S1): Uncropped original blots for figure 1 Western blot. A &B.** Expression profile of ADK-L and ADK-S proteins in the cerebellum from Adk-tg and WT mice at different postnatal developmental stages. Representative blots show ADK (L and S) isoform expression at the cerebellar developmental stages P5, P7, P9, P15, P21, with the long ADK-L isoform appearing as an upper band and the short ADK-S isoform as the lower band. The WT cerebellum showed increased expression of both ADK isoforms (S and L) during developmental maturation. In contrast, the Adk-tg cerebellum was characterized by constant expression of the *Adk-S* transgene at all developmental stages.


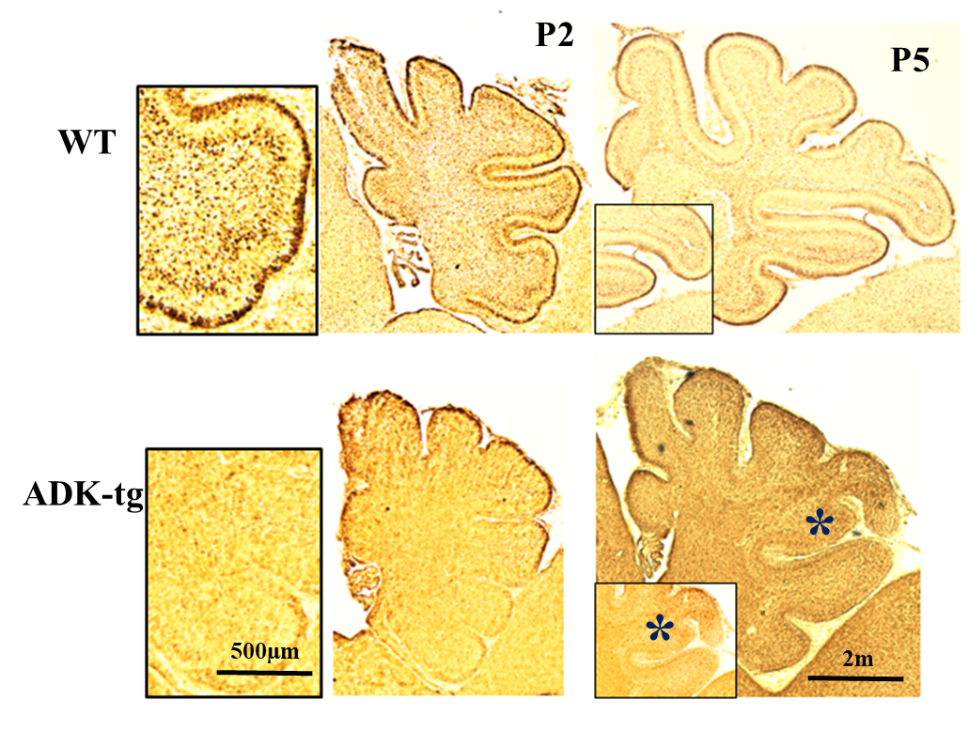


**Supplementary Figure** **2 (S2):** 3,3′-diaminobenzidine (DAB) immunoreactivity of ADK in ADK-tg and WT mice at P2 and P5. In WT, ADK is caracterized by intense expression in the nuclie of cells in the external granule layer (EGL), the molecular layer (ML), and internal granular layer (IGL) at early developmental stages (P2 and P5) in WT. In ADK-tg, ADK expression is characterized by diffused cytoplasmic expression in all layers of the cerebellum and at all developmental stages. Black asterisk label irregular folding in ADK-tg at P5.


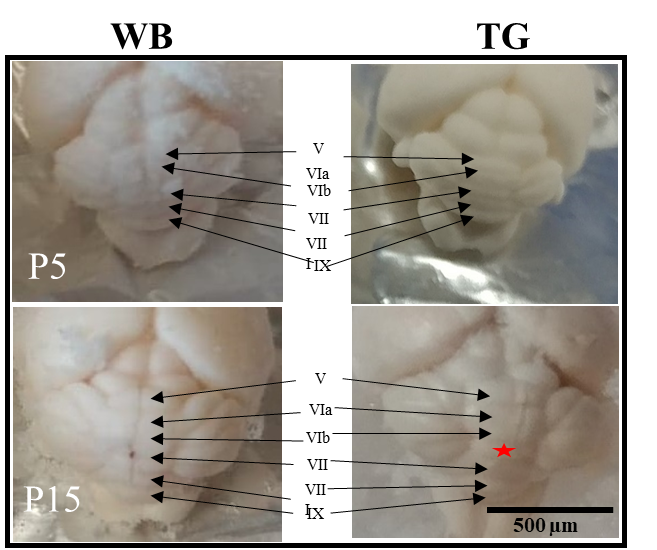


**Supplementary Figure** **3 (S3):** Whole cerebellum picture of Adk-tg and WT mice at P15. In P15, an additional fold exists in the Adk-tg cerebellum, as labeled by a red asterisk. Scale bar is 500 µm.
